# General Principles for the Validation of Proarrhythmia Risk Prediction Models: An Extension of the CiPA *In Silico* Strategy

**DOI:** 10.1002/cpt.1647

**Published:** 2019-11-10

**Authors:** Zhihua Li, Gary R. Mirams, Takashi Yoshinaga, Bradley J. Ridder, Xiaomei Han, Janell E. Chen, Norman L. Stockbridge, Todd A. Wisialowski, Bruce Damiano, Stefano Severi, Pierre Morissette, Peter R. Kowey, Mark Holbrook, Godfrey Smith, Randall L. Rasmusson, Michael Liu, Zhen Song, Zhilin Qu, Derek J. Leishman, Jill Steidl‐Nichols, Blanca Rodriguez, Alfonso Bueno‐Orovio, Xin Zhou, Elisa Passini, Andrew G. Edwards, Stefano Morotti, Haibo Ni, Eleonora Grandi, Colleen E. Clancy, Jamie Vandenberg, Adam Hill, Mikiko Nakamura, Thomas Singer, Liudmila Polonchuk, Andrea Greiter‐Wilke, Ken Wang, Stephane Nave, Aaron Fullerton, Eric A. Sobie, Michelangelo Paci, Flora Musuamba Tshinanu, David G. Strauss

**Affiliations:** ^1^ Division of Applied Regulatory Science Office of Clinical Pharmacology Office of Translational Sciences Center for Drug Evaluation and Research US Food and Drug Administration Silver Spring Maryland USA; ^2^ Centre for Mathematical Medicine & Biology School of Mathematical Sciences University of Nottingham Nottingham UK; ^3^ Global Cardiovascular Assessment Eisai Co., Ltd. Tokyo Japan; ^4^ Division of Biometrics VI Office of Biostatistics Office of Translational Sciences Center for Drug Evaluation and Research US Food and Drug Administration Silver Spring Maryland USA; ^5^ Division of Cardiovascular and Renal Products Office of Drug Evaluation I Office of New Drugs Center for Drug Evaluation and Research US Food and Drug Administration Silver Spring Maryland USA; ^6^ Global Safety Pharmacology Pfizer, Inc. Groton Connecticut USA; ^7^ Global Safety Pharmacology/Nonclinical Safety Janssen Research & Development Raritan New Jersey USA; ^8^ Department of Electrical, Electronic, and Information Engineering University of Bologna Cesena Italy; ^9^ Department of Safety Assessment and Laboratory Animal Resources Merck & Co. Kenilworth New Jersey USA; ^10^ Main Line Health and Sidney Kimmel Medical College Philadelphia Pennsylvania USA; ^11^ Certara, Ltd. Sheffield UK; ^12^ Clyde Biosciences Ltd Glasgow UK; ^13^ Institute of Cardiovascular and Medical Sciences Glasgow University Glasgow UK; ^14^ Department of Physiology and Biophysics Jacobs School of Medicine and Biomedical Sciences University at Buffalo Buffalo New York USA; ^15^ Division of Cardiology Department of Medicine University of California, Los Angeles Los Angeles California USA; ^16^ Department of Toxicology and Pathology Eli Lilly and Company Indianapolis Indiana USA; ^17^ Covance Inc. Madison Wisconsin USA; ^18^ Department of Computer Science University of Oxford Oxford UK; ^19^ Department of Pharmacology University of California Davis Davis California USA; ^20^ Victor Chang Cardiac Research Institute Darlinghurst New South Wales Australia; ^21^ St Vincent's Clinical School UNSW Sydney Darlinghurst New South Wales Australia; ^22^ Clinical Pharmacology Department Chugai Pharmaceutical Co., Ltd. Tokyo Japan; ^23^ Roche Pharma Research and Early Development Pharmaceutical Sciences Roche Innovation Center Basel F. Hoffmann‐La Roche Ltd. Basel Switzerland; ^24^ Roche Product Development, Safety Science/Licensing & Early Development F. Hoffmann‐La Roche Ltd. Basel Switzerland; ^25^ Department of Safety Assessment Genentech, Inc. South San Francisco California USA; ^26^ Icahn School of Medicine at Mount Sinai New York New York USA; ^27^ BioMediTech Faculty of Medicine and Health Technology Tampere University Tampere Finland; ^28^ Federal Agency for Medicines and Health Products Brussels Belgium; ^29^ European Medicines Agency Amsterdam The Netherlands

## Abstract

This white paper presents principles for validating proarrhythmia risk prediction models for regulatory use as discussed at the *In Silico* Breakout Session of a Cardiac Safety Research Consortium/Health and Environmental Sciences Institute/US Food and Drug Administration–sponsored Think Tank Meeting on May 22, 2018. The meeting was convened to evaluate the progress in the development of a new cardiac safety paradigm, the Comprehensive *in Vitro* Proarrhythmia Assay (CiPA). The opinions regarding these principles reflect the collective views of those who participated in the discussion of this topic both at and after the breakout session. Although primarily discussed in the context of *in silico* models, these principles describe the interface between experimental input and model‐based interpretation and are intended to be general enough to be applied to other types of nonclinical models for proarrhythmia assessment. This document was developed with the intention of providing a foundation for more consistency and harmonization in developing and validating different models for proarrhythmia risk prediction using the example of the CiPA paradigm.

In July 2013, a Think Tank jointly sponsored by Cardiac Safety Research Consortium (CSRC), Health and Environmental Sciences Institute (HESI), and the US Food and Drug Administration (FDA) proposed a new cardiac safety paradigm, Comprehensive *in Vitro* Proarrhythmia Assay (CiPA). CiPA uses a new mechanistic, model‐informed approach to predict the risk of Torsade de Pointes (TdP), a rare but potentially lethal form of ventricular tachycardia that can be induced by drugs and lead to sudden death.^1^ Since its inception, global stakeholders including regulatory agencies (the FDA, European Medicines Agency, Health Canada, and the Japan Pharmaceuticals and Medical Devices Agency), industry, and academia have assembled various working groups under a joint effort, the CiPA Initiative, to drive forward different components of CiPA.^2^, ^3^, ^4^ Relevant to this white paper, the *In Silico* Working Group (ISWG) has developed^5^, ^6^, ^7^, ^8^ and validated^9^ an *in silico* cardiomyocyte model that can be used as part of an integrated TdP risk prediction tool under the new CiPA paradigm.

To evaluate the progress of the CiPA Initiative, CSRC/HESI/FDA sponsored another Think Tank meeting in Washington, DC on May 21, 2018,^10^ which was followed by a series of breakout sessions for in‐depth discussion of various components of CiPA. This white paper presents the outcome of the *In Silico* Breakout Session, which primarily focused on establishing a set of general principles to qualify models/metrics to be used for proarrhythmia risk prediction using the example of the CiPA paradigm. Although discussed in the context of using *in silico* models to predict TdP risk, the generic nature of these principles makes it possible to apply them to any type of nonclinical models (*in silico*, *in vitro, ex vivo*, and *in vivo*) for the risk assessment of TdP and other types of arrhythmias. Parallel to the ongoing Q&A development process to modify the current International Council for Harmonisation (ICH) cardiac safety regulatory guidelines,^11^ the development of this document is intended to provide a starting list of consideration points for stakeholders to evaluate and compare different and newer proarrhythmia risk prediction models to subsequently aid in decision making.

## Background

The CiPA paradigm was proposed to address the lack of specificity of the current cardiac safety paradigm that relies on surrogate markers of *in vitro* hERG block and *in vivo*/clinical QTc prolongation,^1^ which are stipulated by the ICH S7B^12^ and E14 guidelines,^13^ respectively. Although highly successful in preventing new drugs with potential TdP risk from entering the market, the current paradigm is considered to have low positive predictive value^10^ and may have unduly constrained drug development.^2^ In recognition of these limitations, the industry and scientific community have developed and characterized a plethora of additional assays (*in vitro* patch clamp assays on other ion channels,^14^ induced pluripotent stem cells (iPS)‐derived cardiomyocytes,^15^ *in silico* model‐based assays,^16^ etc.) with the aim of improving our understanding and management of proarrhythmia risk. The CiPA Initiative represents a community‐wide effort to develop such an assay by taking a focused, systematic, and mechanistic approach to integrate various components. Under CiPA, the assessment of TdP risk for new drugs will be based primarily on mechanistic *in silico* cardiac electrophysiology models parameterized by *in vitro* measurements of drug effects on various cardiac ion channels, with a check of additional effects that may be missed by *in vitro*/*in silico* assays using integrated biological systems (human electrocardiography and stem‐cell‐derived cardiac myocytes).^2^ Because the intended use of *in silico* proarrhythmia risk prediction models under CiPA is aiding regulatory decision making, a rigorous approach to strictly separate model training from validation is preferred,^17^ as discussed in regulatory guidelines about biomarker qualification.^18^ Of note, the term "validation" is intended here as the evaluation of “how good is the model for a given prediction task” rather than of “how good is it as a representation of the real physiological system,” although the latter is also important for model development (see Principle 5 below). The term proarrhythmia risk prediction model in this document refers to the entire prediction system that includes the underlying platform to mimic the response of a physiological system (could be *in silico* models or *in vitro*/*in vivo*/*ex vivo* experimental models) as well as the associated prediction metrics and scoring algorithms for risk assessment.

The CiPA Steering Committee proposed^2^ a development pipeline with a step‐wise approach that includes model training using a set of 12 training drugs, model freezing with prespecified performance measures and acceptable levels, and finally model validation using a dedicated set of 16 validation drugs (see ref. 19 for a review of this strategy). It is envisioned that a similar prospective design could serve as the general framework for qualifying any nonclinical models (*in vitro*, *in silico*, *ex vivo*, *in vivo*) for proarrhythmia risk prediction. However, a set of principles needs to be established to ensure that different models subjected to this validation framework are evaluated in a consistent manner.

Principles for validating specific types of computational models have been established and adopted by international regulatory guidelines in other areas. In 2004 a set of principles for the validation of (quantitative) structure–activity relationship (QSAR) models for regulatory purposes was established by the Organization for Economic Co‐operation and Development (OECD), an intergovernmental organization to coordinate and harmonize policies across member countries.^20^ These principles were later fully adopted by ICH guideline M7(R1) to evaluate the acceptability of QSAR models for the regulatory assessment of mutagenic impurities in pharmaceuticals.^21^ Although OECD principles were specifically developed for QSAR models, they provide a conceptual framework that can be generalized to other types of risk prediction models. In addition, the ICH S7B/E14 Q&A Concept Paper^11^ proposes two separate sets of Q&As for S7B: one about experimental protocol standardization and best practice/data quality considerations using various assays (voltage patch clamp, *in vitro* human myocytes, etc.) at the data collection phase, while the other about general principles for proarrhythmia models, which mainly regards evaluating the model's ability to interpret the data and perform risk prediction. Such a conceptual separation makes it possible to discuss generic rules of evaluating the predictivity of risk prediction models without specifying the underlying assays for data collection and type of collected data. Since the OECD principles provide a prototype for such generic rules, the principles discussed at the *In Silico* Breakout Session of the CiPA Meeting were primarily based on the OECD principles, with the context realigned with the purpose of proarrhythmia risk assessment. This new set of CiPA principles are discussed in the following Basic Principles section.

## Basic Principles

### Principle 1: A defined end point consistent with the context of use

This principle is modified from OECD principle 1,^20^ with the added emphasis that the end points of model predictions should be consistent with the “context of use” (CoU). The end point of a proarrhythmia risk prediction model defines what type of (proarrhythmia) risk it predicts, and the CoU defines the role and scope of a model in addressing specific questions or making specific decisions.^22^ For example, if the defined end point is the risk of drug‐induced TdP, then one possible CoU could be to use the model prediction as part of an integrated risk assessment to determine appropriate language in the drug label about its TdP risk.^11^ Such an end point needs to be defined using a clinical risk categorization system (a set of drugs with known clinical TdP risk liabilities and known clinical exposure levels).^1^ It is possible to use other types of risk as model end points. For instance, QT prolongation is an end point closely related to but distinct from TdP, despite the fact that some clinical categorization systems mix these two end points.^23^ Models designed to predict the risk of QT prolongation should have the end point defined by a series of drugs' clinical QT information, such as Gintant *et al*.^24^ categorization system of QT positive vs. QT negative drugs based on Thorough QT studies. In addition, *in silico* models have been developed to simulate various types of proarrhythmia, including ventricular tachycardia.^25^, ^26^ Once appropriate clinical risk categorization systems are established, these end points can be used by the *in silico* models to predict the risk of different types of drug‐induced proarrhythmia.

Numerous clinical TdP risk categorization systems exist in the literature. For example, Redfern *et al*.^27^ developed a five‐class TdP risk categorization system that is widely used, and subsequently updated/expanded by various groups.^28^, ^29^, ^30^ Arizona Center for Education and Research (AZCERT) maintains Web‐based lists of drugs that are categorized into classes with known, possible, or conditional risk of QT prolongation and/or TdP.^23^ Primarily based on these databases, a number of two‐class categorization systems (TdP+ and TdP−) have also been proposed.^31^, ^32^ To address limitations from some of the above systems (e.g., a mixture of QTc prolongation risk with TdP risk creating a mismatch between end points and CoU), the CiPA Initiative organized a team of expert clinicians, safety pharmacologists, and cardiac electrophysiologists to develop a dedicated CiPA system where 28 drugs were categorized into high, intermediate, and low risk of TdP based on public literature, the FDA adverse event reporting system (AERS) database, and expert opinion.^2^ It is generally recognized that true risk levels are likely to be on a continuous scale rather than discrete.^33^ At present it is difficult to define such a continuum of clinical TdP risk for reference drugs due to the lack of reliable quantification of each drug's risk level. The CiPA three‐class system represents a compromise between quantitative and qualitative risk assessment by classifying reference drugs discretely into ranked risk levels (High > Intermediate > Low). Regulatory decision making depends on benefit–risk assessment.^34^ A more fine‐grained risk classification (such as the classification of High vs. Intermediate risk instead of a single High risk category), although not as informative as a continuous risk quantification system, might help to distinguish drugs with similar benefit profiles but different benefit–risk ratios. For instance, if two drugs have different risk levels (one high and one intermediate risk) but similar benefit (the same indications with comparable efficacy), then the one classified as intermediate risk could be a better opportunity for development compared with the high risk one. On the other hand, if two drugs have the same risk levels (both intermediate) but different benefit (one with less dangerous alternative and one without), then the one with readily available alternatives might be treated with more caution. As an extreme example, astemizole and some other intermediate risk drugs were removed from the market due to such considerations.^35^

Because of the rarity of TdP and complex confounding factors like comorbidity and comedication, at present there is no consensus clinical TdP risk categorization system, and many models were developed using different TdP risk categorization systems with different drug sets and sometimes contradicting risk level assignments.^36^ Ideally, a progressive harmonization of existing TdP risk categorization systems should take place that undergoes a public and transparent process to generate a large set of drugs with consensus clinical risk categories. At present, individual stakeholders should evaluate existing clinical proarrhythmia risk categorization systems, such as the CiPA categories for TdP risk, and decide on the best calibration system that fits the specific purpose.

### Principle 2: An unambiguous algorithm

This principle is the same as OECD Principle 2.^20^ Predicting clinical proarrhythmic risk using a model‐informed approach is a multistep process that involves a series of individual algorithms. For example, some published *in silico* cardiac models for TdP risk prediction usually include a cardiac cellular model to reconstruct electrophysiology,^16^, ^37^, ^38^ a pharmacological component to translate *in vitro* ion channel block data into pharmacological parameters (either half inhibitory concentrations (IC50s) or dynamic drug binding parameters,^7^) and a specific simulation protocol to compute predictive TdP risk metric(s), such as APD90,^29^ qNet,^9^ APD50 plus diastolic calcium,^32^ repolarization abnormalities,^39^, ^40^, ^41^, ^42^ or threshold concentration to cause TdP.^43^, ^44^ In addition, there are also studies where *in vitro* ion channel data were directly input into simpler statistical models to derive a TdP risk metric.^31^, ^45^, ^46^ Finally, even *in vitro* and *in vivo*/*ex vivo* experimental models, such as the iPS cell‐derived cardiomyocyte (iPS‐CM) assays developed by Japan iPS Cardiac Safety Assessment (JiCSA),^15^, ^47^ and canine Purkinje fiber models,^30^ require computational algorithms to integrate various electrophysiological measurements into a compound scoring system for TdP risk prediction. For example, the JiCSA study evaluated two algorithms for risk classification. In the first algorithm, a TdP risk score based on the maximum changes of field potential duration induced by drugs in the iPS‐CM assay is combined with a margin value (the ratio of human free therapeutic plasma concentration to the free concentration in culture medium at which an arrhythmia event was observed) to form a two‐dimensional map to classify drugs into three risk categories.^15^ In the second algorithm, a logistic regression model developed by the CiPA international multisite iPS‐CM study^48^ was applied to the JiCSA data set,^49^ which combines the type of arrhythmia event at any concentration, the maximum repolarization change at any concentration, and the amount of repolarization change at maximum free therapeutic concentration induced by a drug on iPS‐CM assays to perform risk classification. Although different in format (a decision tree based on visual separation vs. a statistical model), both are clear and transparent algorithms to integrate and interpret underlying electrophysiological measurements for risk classification.

It is expected that all models to be used under CiPA will need to have their algorithms and both training and validation data fully disclosed, to ensure transparency and enable the users and other interested parties to reproduce the results during model development and reevaluate the model performance.

### Principle 3: A defined domain of applicability

This principle is essentially the same as OECD Principle 3,^20^ with the underlying concepts redefined to fit the purpose of evaluating models that integrate experimental data for risk prediction. A domain of applicability defines the scope and limitations of a model based on the information contained in the development (training and validation) data sets, and a given model can only be expected to make reliable predictions within the applicability domain, or in a “similar” situation to the development data set. Since a model under CiPA utilizes experimentally derived pharmacological effects for risk prediction, its applicability domain is defined by the experimental procedures applied to, as well as pharmacodynamic effects associated with, the drugs during model development.

This principle has several implications. The first one is that during the model development stage, all drugs should be tested with the same experimental protocol to establish a uniformly defined applicability domain. Note that these protocols can differ for different ion channels, but each drug will need to be tested under the same experimental conditions for the same channel. This is especially important because estimates of drug potency (e.g., IC50) are highly dependent on the patch clamp experimental methods used, and differences in voltage protocols, temperatures, ion channel expression systems, and quality control standards can lead to very different potency estimates across laboratories.^9^, ^50^, ^51^, ^52^, ^53^, ^54^ The second implication is that data used for any new compound predictions by a given model will need to be generated using the same experimental protocols as those used for the model training and validation compounds. Otherwise, the new drug may be outside of the applicability domain of the model, and thus reliability of predictions cannot be established.

Another implication for this principle is that any proarrhythmia risk prediction model can only cover a limited number of proarrhythmic mechanisms, which also define the applicability domain of the model. A prediction could be unreliable even when the new drug follows the same experimental and analytical processes as those during model development, when the new drug has a proarrhythmic mechanism not considered by the proposed model, for instance effects on the beta‐adrenergic system, hERG trafficking, phosphoinositide 3‐kinase pathway,^55^ or blocking effects on ion channels not included in the experimental assay. While some of the mechanisms are related to direct ion channel blocking, others are related to long‐term effects that may not have been well studied. Such “missing” effects are intended to be captured by other components of CiPA (e.g., stem cell and/or human electrocardiography) and, if necessary, to help further develop the model and expand its applicability domain.

A special case about changed applicability domain due to changed pharmacodynamics is that *in silico* models simulating drug actions on normal human cardiomyocytes could be modified to simulate drug actions on specific patient populations or disease situations. Such domain expansion could be achieved through two approaches:^56^ (i) create an ensemble of models with different parameter sets to represent a population of “virtual patients” with varying electrophysiological parameters such as ion currents conductance;^57^, ^58^, ^59^ and (ii) re‐fit (adjusting the parameters and/or structure of) the baseline model to represent a specific clinical situation, such as long QT syndromes.^60^, ^61^, ^62^, ^63^ These are useful methods that may enable risk assessment at the subpopulation or even individual patient level. However, to validate such predictions, reliable end points (clinical risk categorization) would need to be available for such subpopulations (please see Principle 1 above and Principle 4 below). In addition, appropriate CoU would need to be established to use these models on specific subpopulations (Principle 1).

Other than different proarrhythmic mechanisms/pharmacodynamics, the most common cause for drugs falling outside of the applicability domain, despite use of the same experimental procedures, comes from lab‐to‐lab variabilities. These variabilities may stem from subtle experimental factors that are hard to standardize across labs, e.g., cell lines and culture conditions; or they could be due to inherent randomness and intrinsic variability during experimental recordings.^51^, ^64^, ^65^ Under CiPA, an effort is ongoing to standardize not only experimental protocols but also quality control criteria across labs, with the aim of reducing lab‐to‐lab variabilities in generating ion channel pharmacology data. Before such a standard is fully developed and implemented, due to variability in ion channel pharmacology data obtained across labs, it will be necessary for each lab to establish its own applicability domain for a given model. To reduce redundant work, it is recommended that, after a proarrhythmia model has gone through the training and validation stages using all the compounds in the development set, a strategy could be established for each model to select a subset of “calibration” drugs. This set of drugs would allow each individual laboratory to check the ability of its internal experimental systems to generate data to inform the model for risk stratification. After performing this lab‐specific validation step, each lab could use this calibration set to establish the classification thresholds tailored to its own data (lab‐specific calibration). Subsequently the lab can submit new drug data, along with newly generated data from control compounds (a predefined and even smaller subset from the “calibration” drugs to assess the consistency between new and historical data), and perform risk predictions based upon its own self‐consistent classification thresholds. Because the purpose of the control compounds is to assess experimental reproducibility in each submission rather than revalidate or recalibrate the model, the number of assays needed for control compounds can be significantly lower than those for “calibration” drugs. As an example, proarrhythmia risk prediction models that use multi‐ion channel (for instance hERG, Cav1.2, Nav1.5 peak and late) *in vitro* data as input may select a number (for instance 8) of drugs as “calibration set.” A lab that wants to use such a model will need to perform 32 ion channel assays (eight drugs × four currents) on the “calibration set” to establish this lab's own classification thresholds. Such lab‐specific validation and calibration is typically performed just once, or repeated only as needed, for each lab. Subsequently for each new drug submission, the lab will need to test one control compound on each specific current, with a total of four control assays (one drug × four currents), to establish reproducibility. The designation of calibration and control compounds, as well as the criteria of a successful lab‐specific validation, is specific to each proarrhythmia risk prediction model and should be part of the model development plan. Since each lab's new drug will be compared with its own historical data (and classification thresholds), the impact of lab‐to‐lab variability could be minimized.

### Principle 4: A stringent strategy and predefined criteria to assess predictivity

This principle is conceptually similar to OECD Principle 4,^20^ but reworded to shift the focus from the careful selection of appropriate statistical methodologies to the stringent design of the validation study. This is because, unlike QSAR models, which are primarily reductionist models relying on selected statistical methods to find patterns from large data sets, CiPA models are usually mechanistic in nature (see Principle 5 below) with extensive information about the underlying biological/pharmacological processes but limited training and validation drug data sets. Thus, the reliability of a particular proarrhythmia risk prediction model lies in the stringency of the design of the validation strategy to assess predictivity.

This consideration prompted the design of a prospective study for the development of TdP risk prediction models by the CiPA Steering Committee.^1^, ^2^ The 28 drugs in the CiPA development set were divided into a training set of 12 and a validation set of 16 compounds, both sets with a diverse distribution of TdP risk categories, drug classes, and electrophysiological characteristics. The calibration of the model and selection of the metric (model training) should be done based on previously published cardiomyocyte data and newly acquired pharmacology data for the 12 training drugs before the collection of *in vitro* data for the 16 validation drugs. Prior to the validation study, the model, metric, classification thresholds, as well as targeted performance levels, are all to be predetermined (“frozen”) based on training data and reported either through publications or time‐stamped prevalidation documents. Such a rigorous design ensures an objective assessment of the predictivity of the model. Of note, such an assessment of predictivity is within specific proarrhythmic mechanisms as defined by the applicability domain of each model (see Principle 3 for details). And the rigor needed for predictivity assessment, reflected as the stringency of predefined acceptable performance criteria, can be determined based on an analysis of the impact and possible consequences of applying the model within its CoU.^22^

This training‐validation strategy is different from the cross validation (e.g., leave‐one‐out‐cross‐validation) method commonly used to validate TdP prediction models.^29^, ^31^, ^32^ This is because a “hidden” validation data set, where the experimental data were not even collected for the validation drugs during the model training stage, provides the highest confidence that the model building and metric selection process cannot use any information from the validation data set. At least one model (CiPAORdv1.0) followed this step‐wise process of selecting the base model,^66^ further calibrating the model and screening for the TdP metric based on training data,^5^, ^6^, ^7^ and successfully passed the validation stage,^9^ suggesting that this prospective design is feasible and can be used to validate proarrhythmia risk prediction models.

A strict separation of the 28 CiPA drugs into a training and a validation set was maintained during the development of CiPAORdv1.0,^9^ not least by not measuring all validation data until training was complete. Such stringency is lost for future models now that all 28 drugs' *in vitro* assessment is finished and data were released into the public domain. Any new models to be developed for TdP risk prediction may need an alternative validation strategy that uses a prospective design. It may be beneficial to apply the CiPA categorization system to additional compounds to get a new list of drugs with clinical TdP risk levels. This new list could be kept from the public and used as a “hidden” validation set to evaluate the performance of new models proposed to be used for regulatory assessment of TdP risk.

A model that successfully went through this training‐validation process is not “frozen” forever. It can still go through further rounds of external validation for the users to understand the limitations and/or gain more confidence of the model. It will also likely have further incremental changes through continued model development after the validation step. Whether or not such further development needs a repeat of the full training‐validation process depends on the nature of the changes. For example, changes to computational methods designed to speed calculations of a validated *in silico* model might only need to show that they provide the same outputs (given the same inputs) as the computational module they are designed to replace and repeat a global evaluation using the old development data set. On the other hand, if a change is applied to an experimental protocol associated with the validated model, then new experimental data may need to be generated for all drugs in the development data set to repeat the training‐validation process. The requirements to maintain the “validated” status of a model after making incremental changes may need to be evaluated on a case‐by‐case basis.

### Principle 5: A mechanistic interpretation

This principle is the same as OECD Principle 5, with the phrase “if possible” removed to emphasize the importance of mechanistic interpretation for CiPA models. Although the term mechanism could be used to refer to both the mechanism of action of a drug (e.g., specific drug‐channel interactions that lead to the pharmacological effects), and the mechanism of arrhythmia (e.g., reentry mechanism), this principle focuses on the first aspect of the term and emphasizes that the model/metric needs to shed light on specific ion channel/biological pathways through which a drug can trigger arrhythmia. As an example, there are two main types of *in silico* models in the literature for TdP risk prediction. One type (such as CiPAORdv1.0^5^) simulates the drug effects on computational models of action potentials at cellular, tissue, or organ levels and outputs a metric related to drug‐induced changes in repolarization to indicate TdP risk.^29^, ^32^, ^39^, ^42^, ^43^, ^61^, ^67^ The second type uses statistical models/machine learning approaches to directly link *in vitro* drug block measurements (IC50, etc.) to the final TdP risk levels.^31^, ^45^, ^46^ Although differing in complexity, the published approaches for both types of models have taken into consideration the pharmacological effects on the two opposing classes of cardiac ion currents (inward and outward currents) that shape the repolarization of action potentials and thus provide some mechanistic insight into drug‐induced TdP. We consider that all the references above do include a mechanistic interpretation (e.g., in even the simplest models cited above, the balance of block of inward and outward currents is an integral part of the approach) and could be used at different stages of drug development. But those machine‐learning algorithms that blindly screened large quantities of measured experimental features, without providing links between ion channels/pathways targeted by the drug and potential arrhythmic influences, would not deliver a mechanistic interpretation.

In addition to providing an explanation of the clinical risk categorization, mechanistic predictions from models add another layer to the validation procedure. Even though the terms “validation” and “mechanism” defined in this document focus on clinical risk predictivity and pharmacology actions, a model needs to represent the fundamentals of the underlying physiological system for a credible prediction. In line with the OECD guidance on QSAR models,^68^ any effort in the validation process to show that a model is consistent with existing knowledge of fundamental biological/pharmacological processes adds to its credibility and provides scientific support for more credible extrapolations than a pure statistical/machine‐learning approach.^69^ This is essentially true since some of the biological processes have been shown to be important for the mechanisms of proarrhythmia through sensitivity analysis.^70^, ^71^ Therefore, the ability of a model to predict a diverse series of data from experiments designed to probe different aspects of the mechanisms underlying proarrhythmia, e.g., time‐dependent changes of hERG currents after drug application, dose response of ion channel block, action potential morphology, can be used to assess the confidence in different models, or guide the further development of existing models,^60^ even if these models have seemingly similar performance in terms of predicting clinical proarrhythmia risk levels in the validation data set.

### Principle 6: Appropriate uncertainty quantification

This principle is not part of OECD Principles and has been added to CiPA Principles to reflect the need to account for uncertainty and variability to make informed decisions using quantitative models. CiPA models rely on experimentally derived pharmacological parameters as model input to predict clinical proarrhythmia risk, but this process has many sources of uncertainty. These uncertainties will need to be captured and “propagated” through the model to quantify the uncertainty in model prediction,^64^, ^72^ which could alter the predicted risk class of a drug. For example, Lazic *et al*.^73^ proposed an illustrative example of using a Bayesian model to predict a drug's QT prolongation risk based on hERG block potency and clinical exposure. In this model various uncertainties were built into and propagated through the model to predict the probability of a compound falling into either the “QT prolongation” or “non‐QT prolongation” class, rather than predict definite binary classification without uncertainty.

One of the major sources of uncertainty is experimental variability in the measurement of pharmacological effects. Real‐world experiments unavoidably exhibit variability,^74^ and in this field, often substantially—by which we mean to a level that can markedly affect a subsequent risk prediction. Because pharmacological parameters (e.g., IC50s) are derived (fitted) from observed data (e.g., dose–response curves), uncertainty quantification typically starts by establishing a distribution of parameters that could have given rise to the experimental data (uncertainty characterization).^64^, ^72^ These probabilistic distributions are then used as model inputs to translate experimental variability into uncertainty of the model output (risk prediction).^5^ Although a large uncertainty in model inputs does not necessarily translate to a large uncertainty in the output due to their complex nonlinear relationship,^5^ we need to quantify this uncertainty to reflect our confidence in both experimental measurements and risk assessment. The concept and quantification strategy for experimental uncertainty in pharmacological measurements applies to any kind of model (*in silico*, *in vitro*, *ex vivo*, and *in vivo*) for proarrhythmia risk prediction.

Another important source of uncertainty is interindividual variability (due to age, gender, ethnicity, etc.) in the subjects to whom drugs are administered in the real world, which leads to variability in the effects that the very same plasma drug concentration can have on each subject. This can be treated as variability in the physiological parameters of those *in silico* models that simulate action potentials (as, for example, in ref. 39). While some of these parameters are directly measurable, most of them have to be indirectly inferred from experimental data,^65^ similar to pharmacological parameters. Thus, the probabilistic distribution method mentioned above could in theory be applied as well. However, the sheer number of parameters (tens to hundreds of physiological parameters vs. typically 2–5 pharmacological parameters), and potentially complex covariance structure^56^ may require the development of novel methods to deal with such variability.^65^ Similar methods are being developed in other areas (such as physiologically‐based pharmacokinetic modeling/PBPK) for realistic characterization of population variability in a probabilistic framework.^75^ In addition, there are many other sources of uncertainty, such as the uncertainty in model structures as well as numerical precision.^74^ Typically, there is a tradeoff between biological plausibility (including more sources of uncertainty to reflect reality) and model parsimony (using a less complex model).

Note that any sources of uncertainty a model intends to include must be defined clearly as part of the algorithm (Principle 2) and be included in the training and validation processes (Principle 4). Of all the different sources of uncertainty, at a minimum, models should characterize and quantify the uncertainty from experimental variability in measurements of pharmacological effects, as such variability captures the uncertainty in measuring a drug's inherent pharmacological properties (such as IC50s in ion channel assays, drug‐induced arrhythmia events in iPS cell‐derived cardiomyocyte assays^48^, and drug‐induced action potential changes in isolated Purkinje fibre assays^30^) and forms drug‐specific inputs to any kind of proarrhythmia risk prediction models *(in silico*, *in vitro*, *ex vivo*, or *in vivo*).

## Summary and Recommendations

**Figure 1:**
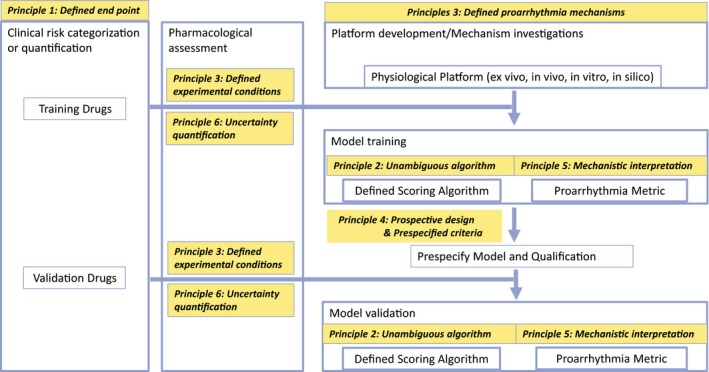
A generic flowchart of proarrhythmia risk prediction model development process. Different principles are applied to different steps along the model development process. In the first step, a defined end point (type of proarrhythmia risk) consistent with the context of use (**Principle 1**) is chosen. This is enabled by testing a list of drugs based on a defined clinical risk categorization or quantification system. The drug list is split into a training set and a validation set. The training set undergoes some defined pharmacological experimental protocols to generate experimental data as model input (**Principle 3**). The experimental variability in the pharmacological assessment needs to be captured and propagated through the model via uncertainty quantification (**Principle 6**). In the meantime, a platform to mimic the response of physiological system is chosen. Such platforms can be, for example, induced pluripotent stem cell‐derived cardiomyocytes (iPS‐CMs), *in vivo* animals, *ex vivo* tissues, or *in silico* cardiomyocyte models. Such platforms may be subjected to a development process, for instance to adjust the structure and parameters of an *in silico* model to better replicate cardiac electrophysiology, or to induce the differentiation of iPS cells to achieve a more mature phenotype. It is important to characterize the model to determine what proarrhythmic mechanisms such a model can cover (**Principle 3**). The pharmacology data for training drugs can be applied to the developed model to perform model training. Note that sometimes the pharmacology assays are performed directly on the platform (such as iPS‐CM assays), while for other assays these two are separated (such as ion channel data collected by dedicated *in vitro* assays and then applied to an *in silico* model). Either way, data generated from pharmacology assays will be translated through the model using a defined scoring algorithm (**Principle 2**) to generate a proarrhythmia metric that explains the mechanisms of action of the drug to trigger arrythmia (**Principle 5**). After the training step the model and targeted performance criteria are to be prespecified (**Principle 4**), and then the validation drugs are tested in the same pharmacology assays to generate data for validation. Note that only key steps are shown. Some other aspects, such as continued model development, are left out of this figure for visual clarity.

This white paper presents a series of general principles for the validation of proarrhythmia risk prediction models illustrated by the CiPA paradigm. These principles can be briefly summarized as follows as well as in **Figure** **1**, with detailed explanation and consideration elaborated in the Basic Principles section above.

Generally, a proarrhythmia risk prediction model should have:

1. A defined end point consistent with the context of use, such as the CiPA three‐class TdP risk categories, or a progressive harmonization of existing TdP risk categories with an expanded list of reference drugs
2. An unambiguous algorithm, allowing users to reproduce the model development process using associated training and validation data sets and reevaluate the performance
3. A defined domain of applicability, where all drugs are tested using standard experimental procedures and have defined pharmacodynamics of proarrhythmic mechanisms, with a strategy to establish a lab‐specific applicability domain by performing lab‐specific validation and calibration
4. A stringent strategy and predefined criteria to assess predictivity, with a prospective design and step‐by‐step documentation to strictly separate training from validation
5. A mechanistic interpretation of the proarrhythmia risk marker
6. Appropriate uncertainty quantification, at a minimum characterizing and quantifying the uncertainty in the experimentally measured pharmacological effects that are drug‐specific inputs into the models

During the discussion of these principles, a number of future directions were identified as important to develop more accurate risk prediction models for regulatory assessment of proarrhythmia liability. These future directions with high priority include:

1. To harmonize existing clinical risk categorization systems for individual proarrhythmia end points (such as TdP) by generating a large set of drugs with consensus risk quantification (either discrete categories or, more preferably, a continuous spectrum of risk). Specifically, for the end point of TdP risk, although the 28 drugs currently selected by CiPA is a much larger number than was used to support the establishment of the current ICH S7B/E14 guidelines, it is acknowledged that there is a need for larger data sets. The hope is now that with a progressive harmonization of existing TdP risk categorization systems (such as the CiPA 28‐drug system and the JiCSA 60‐drug system) proposed under Principle 1, the number of drugs with consensus TdP risk can be significantly increased in the future, resulting in a much bigger development data set. The challenge of determining the number of additional drugs needed and setting the criteria to select these drugs warrant a public and transparent process to reach consensus.
2. To develop better ways to capture the heterogenous drug response in the population. Population modeling approaches have shown promise in simulating intersubject variability in both general and specific populations, and suggesting which individuals are most at risk of developing proarrhythmia after drug treatment.^39^, ^59^, ^76^ However, there may be a need to develop novel methods to capture the covariance of individual physiological parameters and to integrate the quantification of uncertainty of pharmacological effects with the intersubject variability.

The goal of establishing the principles is to provide a discussion of the baseline guidance for the evaluation of individual proarrhythmia models. Although these principles were illustrated through a few selected examples (especially the CiPAORdv1.0 model), they can be generally applied to any existing or newly developed proarrhythmia risk prediction models for performance assessment. While these principles could help stakeholders (regulatory agencies or pharmaceutical industry) to assess whether a particular risk prediction model could be integrated into their decision‐making process, they are not intended to establish specific criteria or give prescriptive guidance. Nor are these principles comprehensive, as some basic principles that are important for any scientific discipline, such as the development of proper Quality Control and/or Quality Assurance procedures, are left out due to the difficulty in discussing such a complex subject across disciplines (*in silico*,* in vitro*, and *in vivo/ex vivo* models). Instead the objective is to stimulate the formation of a harmonized foundation and conceptual framework for collecting useful information necessary to improve, judge, and compare various models for TdP risk prediction. The primary focus of the described example is evaluating the acceptance of *in silico* models for TdP risk prediction under the CiPA paradigm, but because of their generic nature, these principles could be generally applied to any nonclinical models proposed to be used for proarrhythmia risk assessment. Importantly, models that are developed following these principles will still be combined with other relevant nonclinical and clinical information for an integrative and comprehensive risk assessment.

## Funding

B.J.R. and X.H. are supported by the Research Participation Program at the Center for Drug Evaluation and Research, administered by the Oak Ridge Institute for Science and Education through an interagency agreement between the US Department of Energy and the US Food and Drug Administration. G.R.M. acknowledges support from the Wellcome Trust via a Senior Research Fellowship (grant number 212203/Z/18/Z).

## Conflicts of Interest

As an Associate Editor for *Clinical Pharmacology & Therapeutics*, D.G.S. was not involved in the review or decision process for this paper. P.R.K. has equity interest in Biotelemetry and provides consultations to dozens of companies regarding cardiac safety. Dr. J.S.‐N. is employed by a Contract Research Organization (Covance Laboratories Inc.) that could benefit financially from establishing additional regulatory testing requirements. R.L.R. is the Founder and Part owner of Cytocybernetics Inc., a company that provides ion channel and modeling services related to drug discovery. All other authors declared no competing interests for this work.

## Disclaimer

This report is not an official US Food and Drug Administration guidance or policy statement. No official support or endorsement by the US Food and Drug Administration is intended or should be inferred.
